# Gene expression meta-analysis supports existence of molecular apocrine breast cancer with a role for androgen receptor and implies interactions with ErbB family

**DOI:** 10.1186/1755-8794-2-59

**Published:** 2009-09-11

**Authors:** Sandeep Sanga, Bradley M Broom, Vittorio Cristini, Mary E Edgerton

**Affiliations:** 1 Department of Biomedical Engineering, The University of Texas at Austin, Austin, TX, USA; 2 School of Health Information Sciences, The University of Texas Health Science Center, Houston, TX, USA; 3 Department of Bioinformatics and Computational Biology, The University of Texas M.D. Anderson Cancer Center, Houston, TX, USA; 4 Department of Pathology, The University of Texas M.D. Anderson Cancer Center, Houston, TX, USA

## Abstract

**Background:**

Pathway discovery from gene expression data can provide important insight into the relationship between signaling networks and cancer biology. Oncogenic signaling pathways are commonly inferred by comparison with signatures derived from cell lines. We use the Molecular Apocrine subtype of breast cancer to demonstrate our ability to infer pathways directly from patients' gene expression data with pattern analysis algorithms.

**Methods:**

We combine data from two studies that propose the existence of the Molecular Apocrine phenotype. We use quantile normalization and XPN to minimize institutional bias in the data. We use hierarchical clustering, principal components analysis, and comparison of gene signatures derived from Significance Analysis of Microarrays to establish the existence of the Molecular Apocrine subtype and the equivalence of its molecular phenotype across both institutions. Statistical significance was computed using the Fasano & Franceschini test for separation of principal components and the hypergeometric probability formula for significance of overlap in gene signatures. We perform pathway analysis using LeFEminer and Backward Chaining Rule Induction to identify a signaling network that differentiates the subset. We identify a larger cohort of samples in the public domain, and use Gene Shaving and Robust Bayesian Network Analysis to detect pathways that interact with the defining signal.

**Results:**

We demonstrate that the two separately introduced ER^- ^breast cancer subsets represent the same tumor type, called Molecular Apocrine breast cancer. LeFEminer and Backward Chaining Rule Induction support a role for AR signaling as a pathway that differentiates this subset from others. Gene Shaving and Robust Bayesian Network Analysis detect interactions between the AR pathway, EGFR trafficking signals, and ErbB2.

**Conclusion:**

We propose criteria for meta-analysis that are able to demonstrate statistical significance in establishing molecular equivalence of subsets across institutions. Data mining strategies used here provide an alternative method to comparison with cell lines for discovering seminal pathways and interactions between signaling networks. Analysis of Molecular Apocrine breast cancer implies that therapies targeting AR might be hampered if interactions with ErbB family members are not addressed.

## Background

Gene expression array data can be mined to provide critical insight into our understanding of the relationship between signaling networks and the biology of cancer [1-3]. In addition to identifying individual pathways, recent attention has been given to "cross-talk" or interactions that cause aberrant signaling patterns in cancer [4-6]. The conventional method of identifying oncogenic pathways and their interactions has been through studying cell lines [1,2,7,8]. Our goal is to be able to identify dominant pathways using data mining methods that do not require direct comparison with cell lines.

To pursue our goal we investigate a recently introduced subtype of ER^- ^breast cancer that is hypothesized to result from AR signaling. We analyze the data using several different bioinformatics approaches to pathway discovery. We are able to detect patterns that support the same conclusions reached with comparison to cell lines data by the original authors. In addition, we introduce interactions not previously discovered in the data that have important therapeutic implications. Thus, our results contribute to both bioinformatics and to breast cancer biology.

The ER^- ^breast cancer subtype that we study here has been termed the "molecular apocrine" subtype [8,9] and the "ER^- ^class A" subtype [7] in two separate studies that proposed its existence. The studies were independently performed, but both groups hypothesized AR signaling as a defining feature of the transcript profile, leading us to question whether or not they represent the same tumor subset. One study identifies six of 16 ER^- ^tumors as the molecular apocrine subtype and the other study identifies ten of 41 ER^- ^tumors as the class A subtype. Since there has not been a meta-analysis of both studies to actually confirm that the individual tumor clusters actually represent the same breast cancer subset as defined by gene expression, we start by performing a comparative study. We call this a test of "molecular equivalence," and we propose a set of criteria for establishing molecular equivalence cancer subsets defined by gene expression data: 1) the majority of the molecular phenotype should cluster together and their combined profile should be distinct from the remaining samples in unsupervised clustering of the combined data; 2) there should be significant overlap of the gene signatures used to classify the phenotype from each institution; and 3) a classifier trained on data from one institution should be able to predict the phenotype correctly in the other institution's data, and vice versa. In the process of establishing molecular equivalence, we test different methods of normalizing the data to remove institutional bias and we comment on their effectiveness.

Once having established the molecular equivalence of the group, we use Learner of Functional Enrichment algorithm (LeFEminer), which is based on gene set enrichment [10], and Backward Chaining Rule Induction (BCRI), which is a de novo discovery method [11-13], to identify pathways in the combined data. Both of these methods incorporate existing pathway knowledge from the literature within their methodology. Our results indicate a role for AR in this breast cancer subset. Subsequently, we use a gene expression classifier to identify more molecular apocrine data for discovery of pathway interactions. We use Gene Shaving and Robust Bayesian Network Analysis on this data because it facilitates discovery of interactions that have variable prevalence in the patient population [14,15]. We demonstrate that there are highly prevalent interactions between AR signaling and members of the ErbB family. We discuss the therapeutic implications of cross-talk between AR and members of the ErbB family in molecular apocrine type breast cancer. Taken together, these results demonstrate that data mining methods can be used to generate network information directly from gene expression data.

### Data

The data used in this study were generated on Affymetrix U133A oligonucleotide microarrays and are publicly available [7,8,16-18]. The cohort from Farmer et al. [8] includes 22 ER^- ^breast carcinoma samples with six classified as molecular apocrine. The cohort from Doane et al. [7] includes 41 ER^- ^breast carcinoma samples with ten classified as molecular apocrine. We refer to data generated by Farmer et al. and Doane et al. as the "index cohorts." We use additional cohorts from Ivshina et al. [16], Rouzier et al. [17], and Sotiriou et al. [18], which contain 59, 51, and 34 ER^- ^breast carcinoma samples, respectively, to confirm the existence of the molecular apocrine phenotype in larger cohorts outside the index cohorts and to explore gene network interactions.

## Results

### Data Normalization

**Figure 1:**
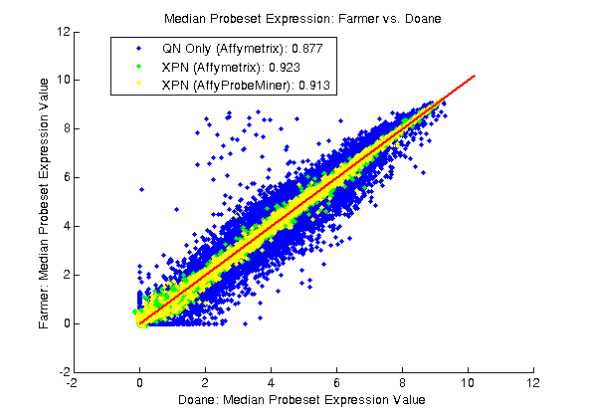
**Scatter plots of Median Probeset Expression Values of Farmer et al. Data vs. Doane et al. Data**. The blue symbols represent the data after quantile normalization (QN), the green symbols represent the data after subsequent cross-study normalization by XPN, and the yellow symbols represent the data after subsequent update of probeset definitions. The red line depicts the unity line, i.e. y = x. The legend lists Pearson correlation coefficient for each normalization step. All data has been natural-log transformed.

We combine the index cohorts into a single, homogeneous dataset with quantile normalization (QN) performed using the dChip software package [19,20] followed by a recently published cross-study normalization scheme (XPN) that results in removal of persistent systematic bias and noise [21]. Additionally, we use updated probeset definitions [22-25]. XPN brings the two gene expression datasets into better agreement as evidenced by improvements in the expected linear relationship of median probeset expression levels between the index cohorts after three sequential steps: 1) QN, 2) QN + XPN, and 3) QN + XPN with updated probeset definitions (Figure 1). The Pearson correlation coefficient for the three steps is 0.877, 0.923, and 0.913, respectively. We note that step 2 gives normalized data with a slightly higher coefficient than with using updated probeset definitions (step 3). However, we choose to follow recommendations in the bioinformatics literature to take advantage of the most up-to-date gene sequence information for grouping and mapping transcript-consistent probesets [22-25]. As we proceed with our analysis, we compare our results using XPN with results we generate using median-centering, a conventional method for cross-study normalization of data performed on a single microarray platform.

### Comparison for Molecular Equivalence

**Figure 2:**
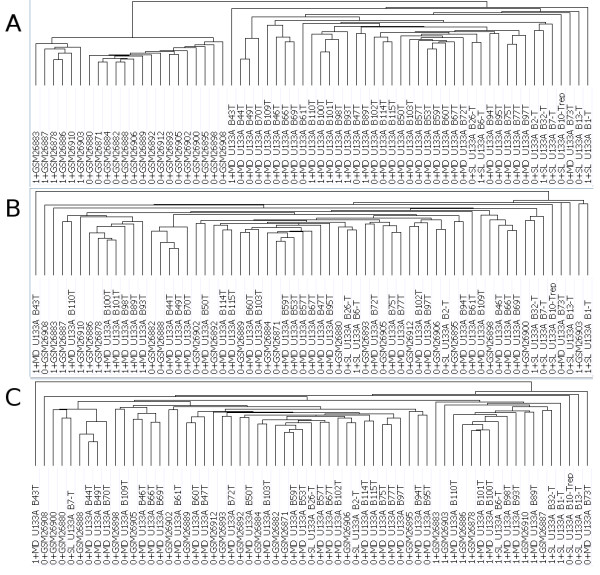
**Hierarchical Clustering of Doane et al. and Farmer et al. Data**. Dendrogram of the clustered data following **(A) **quantile normalization and natural-log transformation by dChip and using original probes sequence information provided by Affymetrix, **(B) **same as **(A) **with added XPN normalization step, and **(C) **same as **(B) **but using updated probe sequence information provided by AffyProbeMiner. Phenotype is denoted by 1 for "molecular apocrine" or 0 for "non-molecular apocrine" preceding the sample ID. Sample id's beginning with GSM were generated by Farmer et al. and otherwise were generated by Doane et al.

**Figure 3:**
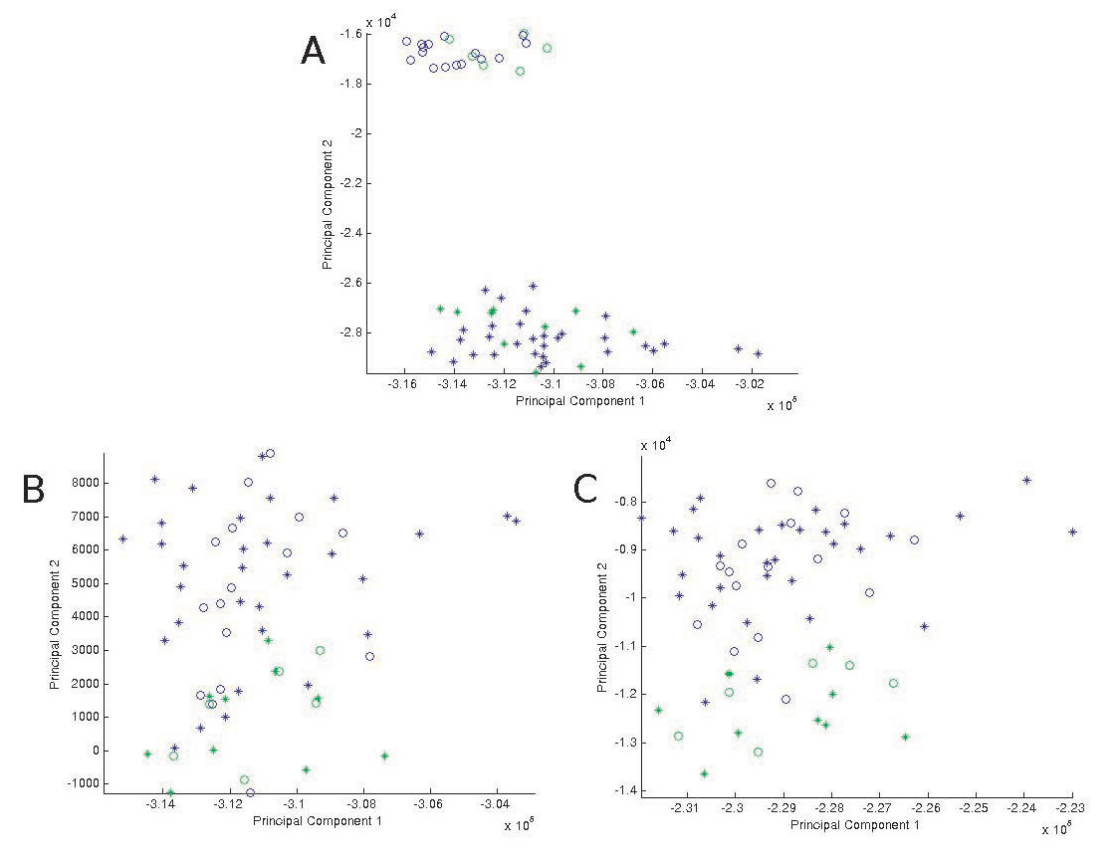
**PCA of Combined Doane et al. and Farmer et al. Cohorts**. First versus second component from Principal Components Analysis (PCA) of the combined cohorts from Farmer et al. (asterisks) and Doane et al. (circles) showing separation of molecular apocrine (green) and non-molecular apocrine (blue) tumors when performed on **(A) **natural-log scaled data quantile-normalized using Affymetrix-provided chip definition file (CDF); p-value = 0.369, **(B) **same as **(A) **with added XPN normalization step; p-value < 0.0001, and **(C) **same as **(B) **but using the AffyProbeMiner-provided CDF; p-value < 0.0001.

When we perform XPN in addition to QN, we see significant improvement in the removal of systematic bias using both unsupervised hierarchical clustering (HC) and principal components analysis (PCA). We use routines in the GenePattern software package [20,26] and separately we compute a p-value using the Fasano & Franceschini statistical test [27]. Figures 2A and 3A demonstrate that the data cluster by institution with QN alone. The addition of XPN to the normalization scheme (Figures 2B and 3B) tapers the institutional systematic bias and reveals a single molecular apocrine cluster. The dendrogram from the HC results shows 12 of 16 (75%) samples defined previously as molecular apocrine in a single cluster (p < 0.0001). Updating the probeset definitions (Figures 2C and 3C) brings the molecular apocrine hierarchical cluster membership to 15 of 16 (94%) samples across the combined index cohorts with improved separation by phenotype (p < 0.0001). We note that median-centering per probeset by institution also results in statistically significant separation (p < 0.0001, see Additional File 1 -Figure S1) with 13 of 16 (81%) molecular apocrine samples clustering together in the HC dendrogram (see Additional File 1 -Figure S2) compared to 15 of 16 (94%) samples using XPN. We note that this difference is not statistically significant.

We evaluate our second proposed criterion for determining molecular equivalence by using Significance Analysis of Microarrays (SAM) [28] to identify the top 100 statistically significant probesets in each of the index cohorts (after normalization) that differentiate the hypothesized molecular apocrine phenotype from the remaining samples. The resulting gene signatures share 76 genes (see Additional file 2), while the original two studies identified 138-gene [7] and 400-gene [8] profiles with 25 overlapping genes. The extent of overlap for both results is statistically significant (both p < 0.0001). For comparison, 100-gene signatures derived from a median-centered dataset using manufacture-provided probeset definitions has 25 overlapping genes and from a median-centered dataset using AffyProbeMiner probeset definitions has 33 overlapping genes (both p < 0.0001). While there is no notable difference in statistical significance, the larger number of common genes gives us more attributes with which to investigate the networks and gene interactions that define this species.

**Figure 4:**
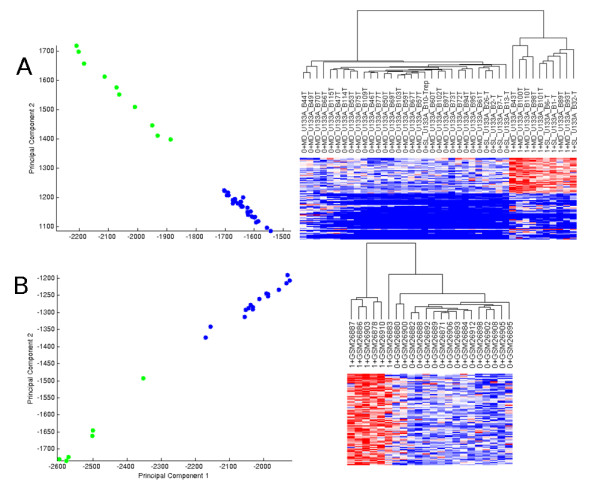
**PCA and Hierarchical Clustering of One Cohort using 100-Probeset Signature Generated from Second Cohort**. Plots of first versus second component of PCA shows separation of non-molecular apocrine (blue) samples from molecular apocrine (green) samples. Dendrograms denote phenotype as 0 preceding sample number for "non-molecular apocrine" and 1 preceding sample number for "molecular apocrine". Data presented in (A) uses gene signature from Farmer et al to perform PCA and HC on Doane cohort and data presented in (B) uses gene signature from Doane et al to perform PCA and HC on Farmer cohort. All data was log transformed, quantile normalized, processed by XPN, and used AffyProbeMiner CDF.

We perform hierarchical clustering and PCA of the Doane et al. [7] cohort with the 100-gene signature identified with SAM on the Farmer et al. [8] cohort, and vice versa to test compliance with our third criterion (see Figure 4). We compare these results with the performance of the published signatures on the published data as normalized by the submitting institution. While the samples do not group together as tightly (see Additional File 1 -Figures S3 and S4) as they do with the 100-gene signatures derived using our normalized data, the signatures identified by Farmer et al. and Doane et al. can indeed predict the molecular apocrine phenotype in the other cohort without cross-study normalization.

### Functional Analysis of the "Molecular Apocrine" Phenotype Using LeFEminer

Using an approach that builds upon the concept of gene set enrichment, LeFEminer identifies a set of top-ranked gene ontology (GO) categories in the normalized index cohorts (see Table 1). Notably, the "Androgen Up-regulated Genes" (86 genes [29,30]) and "Breast Cancer Estrogen Signaling" (101 genes [30]) GO categories both are identified at 0% false discovery rate, with AR presenting as the top ranked gene in the "Breast Cancer Estrogen Signaling" category; three genes overlap between the two profiles used to define the GO categories. Table 1 shows that the AR and ER signal based pathways are the top two regulatory signaling pathways after metabolic and other enzymatic pathways that are listed. These results support the hypothesis that the molecular apocrine subtype has molecular characteristics of a steroid hormone response similar to that of estrogen response [6,7,31].

**Table 1:** Top-ranking Gene Ontology categories identified by LeFEminer on the normalized index cohorts

Category. Rank	Category. Name	Category. Size
1	carboxylic acid metabolism	138

1	Fatty acid metabolism	47

1	Fatty acid metabolism BioCarta	24

1	MAP00480 Glutathione metabolism GenMAPP	18

5	organic acid metabolism	140

5	aromatic compound catabolism	6

5	MAP00350 Tyrosine metabolism GenMAPP	29

8	oxidoreductase activity, acting on CH-OH group of donors	44

9	aromatic amino acid family catabolism	5

10	electron transporter activity BioCarta	102

11	Cyclic nucleotide-dependent protein kinase activity	4

**11**	**ANDROGEN UP GENES na**	**56**

13	tyrosine catabolism	3

13	Fatty Acid Synthesis BioCarta	14

15	regulation of locomotion	6

16	acetylgalactosaminyltransferase activity	7

16	polypeptide N-acetylgalactosaminyltransferase activity	7

16	MAP00360 Phenylalanine metabolism GenMAPP	16

**19**	**breast cancer estrogen signalling GEArray**	**92**

20	peroxisome	36

20	MAP00512 O Glycans biosynthesis GenMAPP	7

22	amine catabolism	23

22	cAMP-dependent protein kinase activity	4

24	regulation of cell migration	6

24	microtubule cytoskeleton organization and biogenesis	23

24	mitotic spindle checkpoint	2

24	electron transporter activity	114

24	Microbody	36

29	regulation of behaviour	6

29	neuronal lineage restriction	2

Rank determined by p-value and false discovery rate. All categories shown in table were identified with 0% false discovery rate. The Category. Size column lists the number of genes in the pre-defined dataset representative of the GO category.

### Network Inference Analysis of "Molecular Apocrine" Phenotype Using Backward Chaining Rule Induction and MetaCore

**Figure 5:**
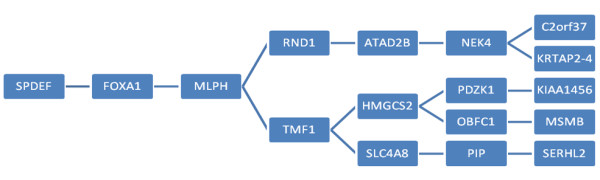
**Network Inference Using Backward Chaining Rule Induction**. The 17 genes discovered by the Backward Chaining Rule Induction strategy applied to the index cohorts.

**Figure 6:**
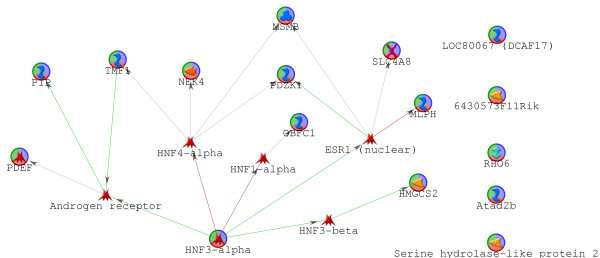
**Prior Knowledge Support for Gene Relationships Identified by Backward Chaining Rule Induction**. Androgen Receptor is closely connected to the 17 genes identified by the Backward Chaining Rule Induction (BCRI) strategy as indicated by using MetaCore to identify the closest paths connecting the genes. Circles superimposed on gene symbols identify the 17 genes from BCRI.

We use See5 as a rule induction method (Rulequest, St. Ives, Australia) and MetaCore, a commercial pathways database with analysis tool distributed by GeneGo (St. Joseph, MI) [32] for implementation of the BCRI strategy for network discovery [11-13]. The method is similar to one previously used to study yeast networks [33]. We followed the BCRI strategy for six successive iterations to identify 17 genes whose expression could predict threshold expression levels of the genes identified in the previous iteration (see Figure 5). A Transcription Regulation Analysis by MetaCore on the 17 genes identifies AR, ESR1 (ER), HNF4-alpha, HNF1-alpha, and HNF3-beta as significant transcription factors regulating the genes identified by BCRI (Table 2). The top 3 regulatory pathways, listed in Table 2, are ER, HNF4-alpha, and AR. In addition, using Dijkstra's algorithm (MetaCore function) to find the shortest known directed paths within two nodes between the 17 BCRI genes results in a network that clearly shows the close relationship between AR and the BCRI genes (Figure 6). The transcription regulation and shortest path analyses (Table 2 and Figure 6) both also specify ER as having a close network relationship with the BCRI genes.

**Table 2:** Transcription regulation analysis by GeneGo's MetaCore

No	Network	GO Processes	Total nodes	Root nodes	p-Value
1	ESR1 (nuclear)	positive regulation of retinoic acid receptor signaling pathway (16.7%; 1.463e-03), negative regulation of mitosis (16.7%; 2.194e-03), epithelial cell maturation (16.7%; 2.559e-03), regulation of retinoic acid receptor signaling pathway (16.7%; 2.925e-03), melanosome localization (16.7%; 4.019e-03)	6	5	3.20E-18

2	HNF4-alpha	negative regulation of protein import into nucleus, translocation (16.7%; 7.318e-04), negative regulation of tyrosine phosphorylation of Stat5 protein (16.7%; 1.463e-03), regulation of protein import into nucleus, translocation (16.7%; 1.829e-03), ornithine metabolic process (16.7%; 2.559e-03), positive regulation of gluconeogenesis (16.7%; 2.559e-03)	6	5	3.20E-18

3	Androgen receptor	prostate gland development (40.0%; 4.905e-06), male somatic sex determination (20.0%; 3.050e-04), somatic sex determination (20.0%; 6.099e-04), gland development (40.0%; 7.323e-04), urogenital system development (40.0%; 7.845e-04)	5	4	1.44E-14

4	HNF1-alpha	glucose homeostasis (66.7%; 5.376e-05), carbohydrate homeostasis (66.7%; 5.376e-05), epithelial cell maturation (33.3%; 1.280e-03), bile acid biosynthetic process (33.3%; 1.646e-03), paraxial mesoderm formation (33.3%; 1.829e-03)	3	2	1.89E-07

5	HNF3-beta	neuron fate specification (66.7%; 2.343e-06), positive regulation of neuron differentiation (66.7%; 6.156e-06), neuron fate commitment (66.7%; 2.409e-05), cell fate specification (66.7%; 3.555e-05), epithelial cell differentiation (66.7%; 6.683e-05)	3	2	1.89E-07

Transcription regulation analysis using GeneGo's MetaCore generates sub-networks centered on transcription factors using the 17 genes discovered by Backward Chaining Rule Induction (Figure 6). Sub-networks are ranked by a p-value and interpreted in based on Gene Ontology (GO) categories.

### Persistence of Molecularly-defined Phenotype in Larger Dataset

**Figure 7:**
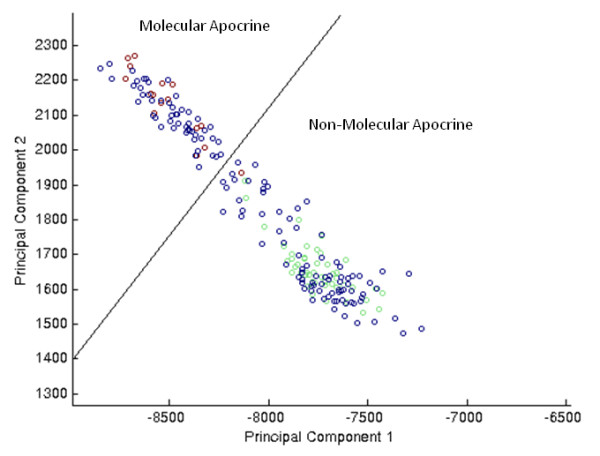
**Principal Components Analysis of Cross-study Normalized, Five-Cohort Dataset**. PCA using 346-probeset signature derived from the combined index cohorts shows that ER^- ^samples from the Ivshina et al., Rouzier et al., and Sotiriou et al. cohorts (blue symbols) associate with either the molecular apocrine (red symbols) or the non-molecular apocrine (green symbols) phenotypes from the index cohorts.

At this point we have supporting evidence for a role for AR in defining the molecular apocrine subtype using two independent methods of network inference. We now seek to identify the gene network and pathways that interact with AR. Limited sample sizes hinder this type of analysis. Therefore, we expand our molecular apocrine gene expression data with ER^- ^samples from Ivshina et al., Rouzier et al., and Sotiriou et al., bringing our total to 199 [7,8,16-18]. We normalize with QN + XPN with updated probeset definitions. We apply SAM to the index cohorts and identify a 346-probeset signature at 0% false discovery rate to predict molecular apocrine samples (see Additional file 3). We use these genes to perform PCA on the expanded cohort. These results show a natural demarcation in the larger ER^- ^dataset where the 22 molecular apocrine sampes in the index cohort along with an additional 46 samples in the expanded cohort separate from the rest of the data (see Figure 7). We refer to these 68 samples as the "model-classifed cohort."

### Network Inference Analysis of "Molecular Apocrine" Phenotype Using Gene Shaving & Robust Bayesian Network Analysis

First, we perform an unsupervised gene clustering using Gene Shaving (GS), and subsequently use Robust Bayesian Network Analysis (RBNA) to discover relationships between an AR-based cluster and other gene clusters [14,15]. Note that we do not seek support for the AR pathway as having a role in the molecular apocrine subtype in the model classified cohort because our gene classifier that predicts membership in the molecular apocrine subtype includes AR. This would have biased the network inferences toward selecting AR.

**Figure 8:**
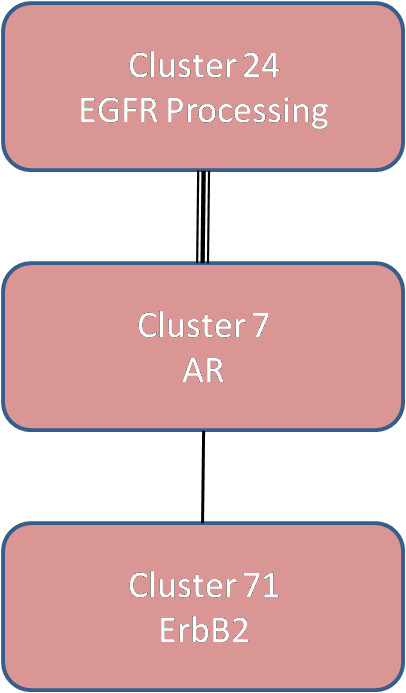
**Robust Bayesian Network Analysis of Top Apocrine-related Gene Clusters Identified Through Gene Shaving Reveals Interactions with AR**. Relative strength of interactions is indicated by bolder links connecting clusters. Cluster 7 (AR cluster) interacts with Cluster 29 (EGFR processing genes) and Cluster 71 (ErbB2 cluster).

We identified the top 200 gene clusters using unsupervised GS (see Additional file 4) ranked according to their internal cluster strength (order of how they were shaved). The cluster containing AR was the 7^th ^ranked cluster (see Additional File 1 -Figure S5). Clusters 24, 29, and 7 were the top three clusters associated with the molecular apocrine phenotype using Kendall's tau log rank analysis (see Methods). We selected RBNA as a network inference method for studying interactions between AR, represented as Cluster 7, and other gene clusters because in addition to discovering relationships between the clusters, it provides a "global" perspective on both *interaction *and *prevalence *in the patient population. The top 26 clusters correlating with the molecular apocrine phenotype analysis were used in this analysis. These clusters were selected using an absolute value of 0.5 as a cut-off for the Kendall's tau log rank. This threshold was defined to maintain sufficient correlation with the molecular apocrine phenotype, to allow clusters to be used which will have interactions that may not involve the entire molecular apocrine phenotype or may overlap with other phenotypes, and finally to provide a sufficient number of clusters for RBNA to sample in order to quantify the relative strengths of interactions in the samples. Network associations amongst 14 of these 26 clusters were identified with RBNA (see Additional File 1 -Figure S6). Figure 8 shows that the AR cluster (see Additional File 1 -Figure S7) is most strongly associated with Cluster 24 (see Additional File 1 -Figure S8) and less so with Cluster 71 (see Additional File 1 -Figure S9). These are the only two clusters that directly interact with the AR cluster, and we select these two for further characterization.

### Analysis of the Interacting Gene Clusters

We submit the members of the interacting clusters to both MetaCore [32] and GeneCards [34,35] to identify associated gene ontologies and known transcription regulation relationships. From GeneCards we identify multiple upregulated species associated with EGFR processing in Cluster 24, which we label as the EGFR processing cluster, although EGFR itself is not a member of the cluster. MetaCore shows that Cluster 71, which contains ErbB2, also contains other EGFR-related genes. We call this cluster the ErbB2 cluster. We also analyzed the clusters that indirectly interact with the AR cluster. Cluster 16 is interesting because although AR is not a member, MetaCore reveals a large number of genes whose transcription is regulated by AR (see Additional File 1 -Figure S10). Furthermore, MetaCore analysis of Cluster 16 suggests network relationships related to ER, p53, and Maspin (a tumor suppressor gene associated with breast, prostate, and pancreatic cancer). In addition to Cluster 16, MetaCore identifies relationships between ER and the genes in the AR and ErbB2 clusters along with clusters 56, 62, 71, 76, 80, and 92. Of interest, ErbB3 is present in cluster 62, which has an indirect link to the molecular apocrine subtype (see Additional File 1 -Figure S6).

## Discussion & Conclusion

Our conclusions are pertinent to both bioinformatics in general and to this particular breast cancer subset.

### Observations of Normalization Strategies to Remove Institutional Bias in Meta-Analysis of Gene Expression Array Data

In the course of our investigation, we compared the effectiveness of normalizing data using quantile normalzation, conventional median-centering, and a recently published algorithm called XPN. Although the data from the two institutions demonstrated adequate correlation after quantile normalization, results of the hierarchical clustering continued to be affected by institutional bias. This may indicate a particular sensitivity of hierarchical clustering to institutional bias.

### Molecular Equivalence of the "ER-Subclass A" with "Molecular Apocrine" Breast Cancer

We have proposed three criteria for evaluating molecular equivalence between transcript-defined subsets identified by two or more independently conducted studies: 1) the majority of molecularly equivalent samples should cluster together and distinctively separate from the remaining samples in unsupervised clustering of the combined data; 2) there should be statistically significant overlap of gene signatures used to define the phenotype in each separate study; and 3) a classifier trained on data from one institution should successfully predict the phenotype in the other institution, and vice versa. We call upon the microarray community to consider these criteria and establish a standard protocol for etablishing molecular equivalence.

In the course of our evaluation, we demonstrate that two of the three criteria proposed are met even without combining and normalizing the data together: the 25-gene overlap between the signatures identified by Farmer et al. and Doane et al. is statistically significant; and the published signatures for each of these studies adequately predicts the hypothesized breast cancer subset in the other index cohort. However, not only were we able to enlarge the extent of overlap in the signatures, but we found that only after appropriate normalization did the samples from the two institutions cluster together by hypothesized phenotype using hierarchical clustering.

### Role of AR Signaling in Molecular Apocrine Tumors

Both authors suggest a role for AR signaling in this subtype of breast cancer based on comparison to data generated by cell lines. In addition, Doane et al. suggests that there is some overlap of the signatures with known ER^+ ^genes. We chose two different network inference methods to explore causal networks in this data. LeFEminer utilizes a gene set enrichment type approach while BCRI functions as a discovery strategy supplemented by pathway information from Metacore. We selected pathways that were common to both strategies as highly supported. The AR and ER signals were the two signaling pathways that were identified by both algorithms as relevant to the molecular apocrine phenotype. Expression of the ER molecular profile in the molecular apocrine group, in spite of the fact that it is ER^- ^by immunohistochemistry, has been described by other authors [6,7,31]. From a bioinformatics perspective, and since BCRI is a relatively new method of network inference, we see this result as validation of its utility in pathway discovery.

### Pathways that Interact with AR in Molecular Apocrine Breast Cancer

Our analysis shows that the molecular apocrine phenotype lacks an overexpression of basal cytokeratins, which is considered to be a defining feature of basal-like breast cancer [36,37]. Thus, we can consider molecular apocrine tumors to be a distinct subset of ER^- ^tumors that includes both triple-negative and ER^-^/PR^-^/ErbB2^+ ^tumors. Since we started our research, two other studies have discovered this subgroup [9,38]. One study identified it within triple-negative tumors alone while the other identified it to combine AR and ErbB2 signaling. We agree with the original authors that the molecular apocrine tumors can be either ErbB2^+ ^or ErbB2^- ^based on intraction studies that we will discuss below.

Our results reveal a strong interaction between the AR cluster and a cluster with several genes involved in EGFR processing. Several cell lines studies have hypothesized an interaction between EGFR and both AR and ER, suggesting that together they form a complex with Src that enhances EGFR phosphorylation of tyrosine and therefore increases the effectiveness of EGF signaling [5,39,40]. However, this is the first study of gene expression data using cancer tissue from patients in which this interaction has been detected using data analysis methods.

A significant relationship is also revealed between the AR cluster and the ErbB2 cluster. The strength of the interaction between this cluster and the AR cluster is weaker than the EGFR processing cluster. In the index studies of molecular apocrine tumors, approximately half of the cases were ErbB2^+^. This is consistent with the less strong, but significant interaction between AR and ErbB2 in our analysis. In addition to simple co-expression, actual cross-talk between ErbB2 and AR pathways has been suggested based on cell line studies in breast [5,6]. These studies demonstrated an additive affect of AR inhibition in reducing ErbB2 signaling, and suggested that tumors that are AR^+^/ErbB2^+ ^might need AR inhibition in addition to targeted anti-ErbB2 therapy to completely neutralize the effective of the ErbB2 signal.

In prostate cancer, cell lines studies have led investigators to hyopthesize that ErbB family signaling, including EGFR (ErbB1), ErbB2, and ErbB3, can activate AR and is responsible for evolution from androgen dependent to androgen independent tumor growth [41]. Thus, at least some tumors with AR transcription profiles might require therapy with ErbB family inihibitors.

Our results with both BCRI and GS combined with RBNA also support the role of FOXA1 interacting with AR in this phenotype. FOXA1 is known to have a role in potentiating steroid receptor transcription regulation, and its association with AR by immunohistochemistry has been reported by several other investigators [42-48]. FOXA1 is a member of the AR cluster and was also directly identified by BCRI (see Additional File 1 -Figure S5). Three other genes identified directly by BCRI (i.e., SPDEF, MLPH, and SERHL) are also part of the AR cluster, which further emphasizes BCRI as a valid network inference strategy.

Associations between PIK3CA mutations and AR in triple-negative tumors have been reported recently [49]. Strong associations between a PIK3CA expressing cluster and AR cluster were not identified. However, given that mutations in PIK3CA may not be picked up on standard gene expression platforms, this association may not be readily discovered from the data.

### Clinical and Therapeutic Implications for Molecular Apocrine Breast Cancer

We propose that therapies targeting AR activity may present a rational strategy for managing these patients. The concept of introducing AR blockade as a therapeutic option for breast cancer has received more attention recently [6-9,50-54]. Older trials of AR blockade did not select for patients with AR dependent signaling or AR expression and therefore may not have addressed the question with an optimal cohort [55]. Based upon our interaction studies, we also recommend that any therapeutic strategies for the molecular apocrine subgroup consider combinatorial targeted therapy to include ErbB family targets, particularly EGFR targeted therapy for the entire molecular apocrine subtype and ErbB2 therapy for those tumors that overexpress ErbB2.

While there is evidence to support ER response genes in the molecular apocrine subset, anti-estrogen therapy using tamoxifen in ER^- ^women in general has been shown to have too little benefit for clinical use. However, small benefits were reported that point to the need for more study [53]. An important question arises - is the presence of ER signaling inferred because AR and ER share a common pathway, or is there cross-talk where AR activation stimulates the ER pathway? Our pathway analyses from BCRI that demonstrate AR and ER as related signals (Figure 6), and analysis of Cluster 16 (see Additional File 1- Figure S10), do not support a common pathway that is activated by AR and ER. While interesting, these results are not conclusive. We note that if cross-talk from activated AR signaling is the cause of the ER signal activation in ER^- ^tumors, then AR inhibition therapy would be sufficient to interrupt this signal.

There is little known about the survival of molecular apocrine tumors as they have only been recently introduced as a subtype. Farmer et al. [8] describes poor survival in the cohort that they identified from the literature. Weigelt et al. [9] suggest that apocrine carinomas can expect a 10-year survival rate of 35-50%, and Teschendorff etl al. [38] suggest that it has the poorest outcome of all of the ER^- ^tumor types. Other data suggests that AR^+ ^tumors that are otherwise triple-negative as defined by immunohistochemistry may have a better prognosis than the basal subtype of tumors [56]. In a recent study of AR protein expression in any type of breast cancer, an improved prognosis was associated with AR expression above a certain threshold in ER^+ ^tumors [49]. It may be that interactions with ErbB family members modify the survival characteristics of AR^+ ^tumors. This deserves further study.

### Learning the Systems Biology of Cancer Using Network Inference Methods to Analyze Gene Expression Data

Our results support the strength of using network inference to analyze gene expression array data for oncogenic pathways and their interactions. This study demonstrates that the discovery of oncogenic pathways and their interactions does not have to rely on comparison with signatures from cell lines, but can be discovered using network inference methods. Thus our results demonstrate the rich knowledge resource within gene expression data generated from human tissues.

## Methods

### Data Collection

The raw CEL files from Farmer et al. [8] are available for download at NCBI GEO Datasets under accession GSE1561. The raw CEL files from Doane et al. [7] are available for download at the National Cancer Institute caArray database. The raw CEL files from Ivshina et al. [16] and Sotiriou et al. [18] are available for download at NCBI GEO Datasets under accession GSE4922 and GSE2990, respectively. The raw CEL files from Rouzier et al. [17] are available for download at http://bioinformatics.mdanderson.org/pubdata.html.

### Microarray Normalization: Removing Systematic and Institutional Bias

The Doane et al. and Farmer et al. cohorts were first quantile-normalized [19] together using the default settings in DNA-Chip Analyzer (dChip), a software package for probe-level analysis of gene expression microarrays [20]. This process was repeated twice: the first time, the original Affymetrix-provided chip definition file (CDF) was used, and the second time, a transcript-consistent Affymetrix-formatted Chip Definition File (CDF) downloaded from AffyProbeMiner [25] was used. A recently published cross-study normalization scheme called XPN [21] was subsequently implemented to further combine the quantile-normalized datasets into a single, unified datasets with significantly reduced systematic bias; one dataset derives from normalization with Affymetrix' CDF and a second dataset (the primary dataset used for analysis in this study) derives from normalization with AffyProbeMiner's CDF. The details regarding the normalization scheme, referred to as XPN, have been previously described [21]. In short, the XPN algorithm is based on linking gene/sample clusters amongst given datasets. Data is scaled and shifted according to the assumption that similar gene-sets cluster together across multiple platforms. XPN has been shown to successfully remove systematic bias, while avoiding the loss of useful biological information due to data over-correction [21].

The other cohorts were included to investigate the persistence of the molecular trends identified in the Doane et al. and Farmer et al. datasets. All five cohorts were quantile-normalized with dChip using a transcript-consistent Affymetrix-formatted CDF provided by AffyProbeMiner [25]. Then, XPN was used in serial increments to bring the five cohorts into uniform agreement by removing persistent systematic bias between the datasets.

### Significance Analysis of Microarrays: Modified T-Test

Significance Analysis of Microarrays (SAM) was performed on the normalized Doane et al. [7] and Farmer et al. [8] data individually to identify top 100 probesets that classify between the molecular apocrine samples and the remaining samples. SAM was also performed on the combined Doane et al. and Farmer et al. subset of the cross-study normalized, five-cohort data to identify a gene signature with 0% false discovery rate for classifying molecular apocrine samples from the remaining samples, and identifying similar molecular trends in the remaining data. SAM is based on a modified T-test; details regarding the algorithm have been previously described [28,57].

### Hierarchical Clustering and Principal Components Analysis

Hierarchical Clustering was performed using a Pairwise-Average Linking method and Euclidian Distance as the distance measure. Both Hierarchical Clustering and Principal Components Analysis were performed on the GenePattern software package provided by the Broad Institute [20,26]. Visualizations of the Principal Components Analysis were performed with MATLAB (Mathworks, Natick, MA).

### Two-Dimensional Kolmogorov-Smirnov Test

The Fasano & Franceschini statistical test [27], a two-dimensional adaptation of the Kolmogorov-Smirnov test [58], was performed on the coordinates derived from the first two principal components using an algorithm provided by Numerical Recipes in Fortran 90 [59].

### Statistical Significance of Overlap Between Gene Signatures

The probability of finding a specified number of overlapping genes between two gene signatures was calculated using the exact hypergeometric probability formula using a web-based tool at http://elegans.uky.edu/MA/progs/overlap_stats.html based on algorithms provided by Numerical Recipes in C [60].

### Backward Chaining Rule Induction

Backward Chaining Rule Induction (BCRI) is a supervised learning approach for identifying relationships amongst genes that can predict for the molecular apocrine phenotype. In order to initialize the BCRI strategy, we use a classifier method called See5 (Rulequest, St. Ives, Australia) to build a prediction model from the normalized gene expression data for classifying the molecular apocrine phenotype from the remaining samples in the index cohorts. Successive iterations of the BCRI strategy infert gene network relationships by predicting threshold expression of genes from other genes. Further details regarding the BCRI strategy have been previously described [11-13].

### Gene Shaving

To identify clusters of highly correlated genes, we used unsupervised Gene Shaving [14]. Specifically, we used a high-performance, parallel C implementation of the method that was developed from the GeneClust software package [61]. Gene Shaving was used independently on both unweighted data and on 127 bootstrap resamples, extracting the first 150 gene clusters in each case. In both cases, the data was first ranked within each sample. To obtain the unweighted data, the ranked data was ranked again, this time across samples within each cohort. For the bootstrap resamples, each sample within a cohort was assigned a random weight chosen from the Bayesian bootstrap distribution [62] and weighted rankings across samples within each cohort were computed. In both cases, the rank of each sample was scaled by the number of samples in the cohort, so that for each cohort the data is in the range zero to one. Robust clusters were obtained from the combined outputs of the Gene Shave runs by selecting those genes that occur frequently together in the outputs of individual runs. We extracted the first 200 clusters with the largest number of co-clustering genes, weighted by the homogeneity of the clusters to which they belong.

### Robust Bayesian Network Analysis

The 200 robust clusters obtained by Gene Shaving were ranked by their correlation with their molecular apocrine phenotype. A cluster meta-gene score was obtained for each sample by computing the signed average mean gene. (Unlike other gene clustering methods, Gene Shaving clusters may include both correlated and anti-correlated genes). The 26 clusters with the highest absolute Kendall Tau correlation between the cluster meta-gene scores and the molecular apocrine phenotype status were selected for network analysis.

The network analysis included nodes for the 26 gene clusters most highly correlated with molecular apocrine status and a node for molecular apocrine status. The cluster meta-gene scores were each discretized to three levels: the lowest, middle, and highest thirds of the expression range for each meta-gene. Forty thousand bootstrap resamples of the discretized weights were obtained by randomly weighting each sample according to the Bayesian bootstrap distribution [62], and a high-scoring network was found for each resample using greedy hill-climbing with random restarts and the sparse candidate algorithm [63]. The scoring function used was DPSM with λ = 1 [64].

Edges that occurred frequently (in either direction) within the forty thousand best networks thus obtained were selected for the final network. Edges that occurred in at least 97.5% of the networks are drawn with a triple black line, those that occurred in at least 95% of the networks with a black line, and those that occurred in at least 85% of the networks with a dashed line. Gene clusters that are not connected by any path along such edges to the node for molecular apocrine status are not included.

## Abbreviations

(AR): Androgen Receptor; (BCRI): Backward Chaining Rule Induction; (EGFR): Epidermal Growth Factor Receptor; (ER): Estrogen Receptor; (ErbB2): Human Epidermal Growth Factor Receptor 2 commonly referred to as Her2neu; (GS): Gene Shaving; (HC): Hierarchical Clustering; (PCA): Principal Components Analysis; (QN): Quantile Normalization; (RBNA): Robust Bayesian Network Analysis; (SAM): Significance Analysis of Microarrays.

## Competing interests

The authors declare that they have no competing interests.

## Authors' contributions

SS carried out the microarray normalization and analysis using SAM, LeFEminer, MetaCore, GeneCards, Hierarchical Clustering, and Backward Chaining Rule Induction. SS also participated in the study design, and drafted the manuscript. BMB carried out the Gene Shaving and Robust Bayesian Network analysis. VC participated in the study design. MEE conceived of the study, directed its design and coordination, and helped to draft the manuscript. All authors read and approved the final manuscript.

## Pre-publication history

The pre-publication history for this paper can be accessed here:

http://www.biomedcentral.com/1755-8794/2/59/prepub

## Supplementary Material

Additional File 1 **Supplementary Figures**. A file containing the supplementary figures S1-S10 referred to in the manuscript. Click here for file

Additional File 2 **The 100-probeset signatures for differentiating molecular apocrine and non-molecular apocrine phenotypes derived from Doane et al. and Farmer et al. cohorts individually**. These gene signatures were derived from the Doane et al. and Farmer et al. samples separately following normalization using log transformation, quantile normalization, XPN processing and updated probeset definitions. There are 76 overlapping genes between the two signatures. The signatures were derived using Significance Analysis of Microarrays. Click here for file

Additional File 3 **The 346-probeset signature for differentiating molecular apocrine and non-molecular apocrine phenotypes**. The signature was identified using Significance Analysis of Microarrays software on the combined Doane et al. and Farmer et al. cohorts at a false discovery rate of 0%. All data was normalized using log transformation, quantile normalization, XPN processing and updated probeset definitions. There are 76 overlapping genes between the two signatures. Click here for file

Additional File 4 **200 Gene Clusters Identified by Unsupervised Gene Shaving**. A list of the genes in each of the top 200 gene clusters identified by unsupervised Gene Shaving. Analysis was performed on all samples (Farmer et al., Doane et al., Ivshina et al., Rouzier et al., and Sotiriou et al.) following normalization using log transformation, quantile normalization, XPN processing and updated probeset definitions. Click here for file
