# Supplementary material for: Gene expression meta-analysis supports existence of molecular apocrine breast cancer with a role for androgen receptor and implies interactions with ErbB family
Source: BMC Med Genomics. 2009 Sep 11;2:59. doi: 10.1186/1755-8794-2-59 (PMC2753593; doi:10.1186/1755-8794-2-59)
Supplement: Additional File 1 — Supplementary Figures. A file containing the supplementary figures S1-S10 referred to in the manuscript. [file 1755-8794-2-59-S1.pdf]

Supplementary Figures S1-S10

Figure S1

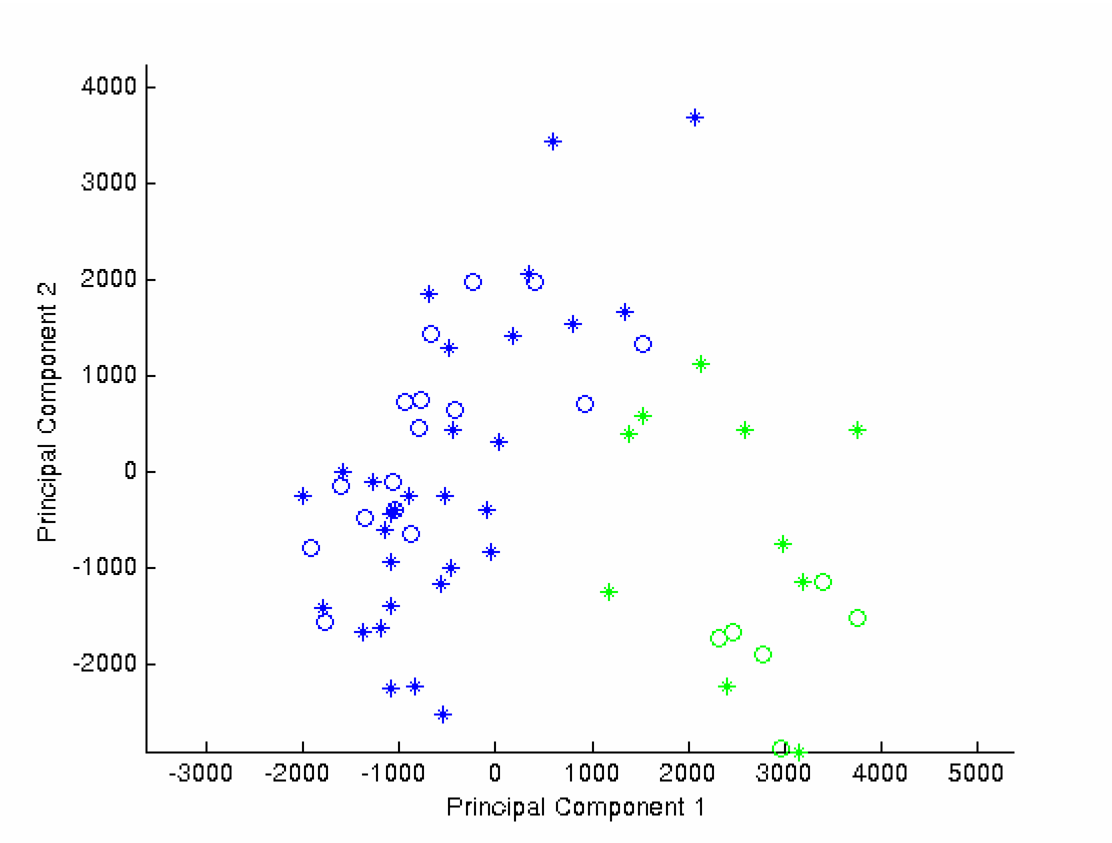

Figure S1: ***PCA of Combined Doane et al. and Farmer et al. Cohorts where Data is Median-Centered.*** Data is natural log-scaled and quantile normalized using the AffyProbeMiner-provided chip definition file (CDF) with median-centering per probeset by institution. Circles represent the Doane et al. cohort and asterisks represent the Farmer et al. cohort. The hypothesized “molecular apocrine” phenotype is represented by the color green and “non-molecular apocrine” phenotype by the color blue.

Figure S2

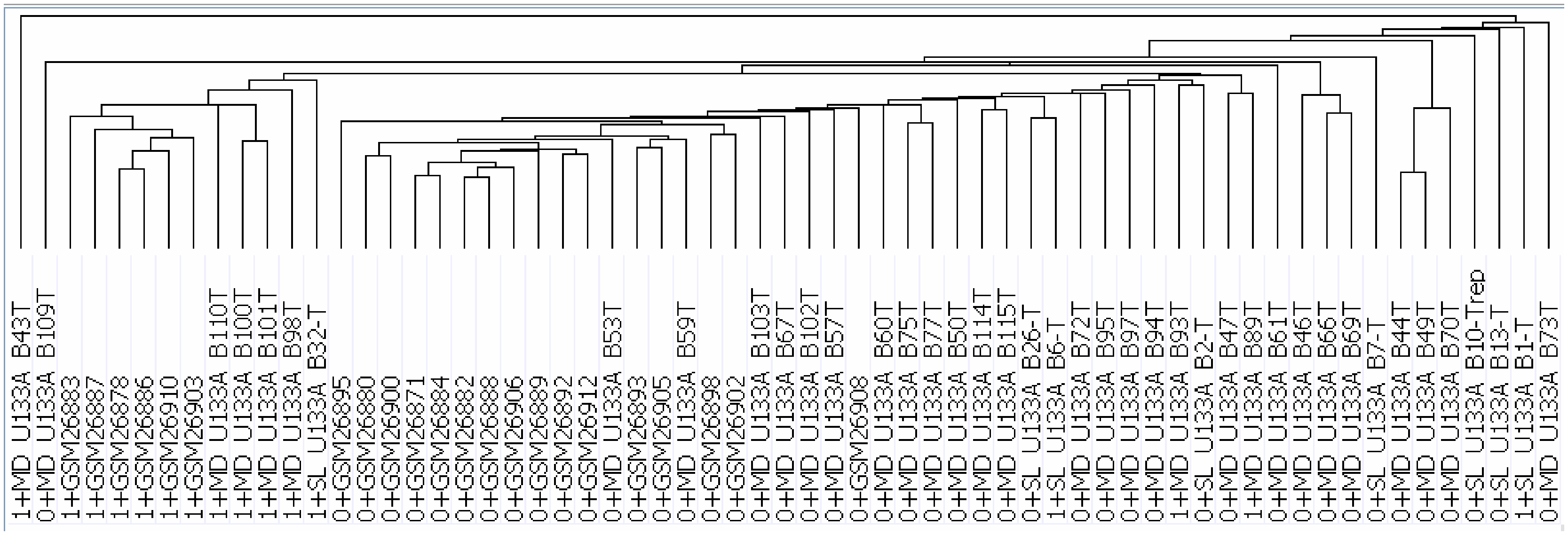

Figure S2: ***Dendrogram after Hierarchical Clustering of Combined Doane et al. and Farmer et al. Cohorts where Data is Median-Centered per Probeset by Institution of the combined Farmer et al. and Doane et al. cohorts.*** Data is natural log-scaled data quantile-normalized using the AffyProbeMiner-provided chip definition file (CDF) with median-centering per probeset by institution. Clustering was performed by Pairwise-Average Linkage using Euclidian Distance. The samples cluster according to institution and not by their “molecular apocrine” or “non-molecular apocrine” phenotype as indicated by 1 and 0, respectively, preceding the sample ID.

Figure S3

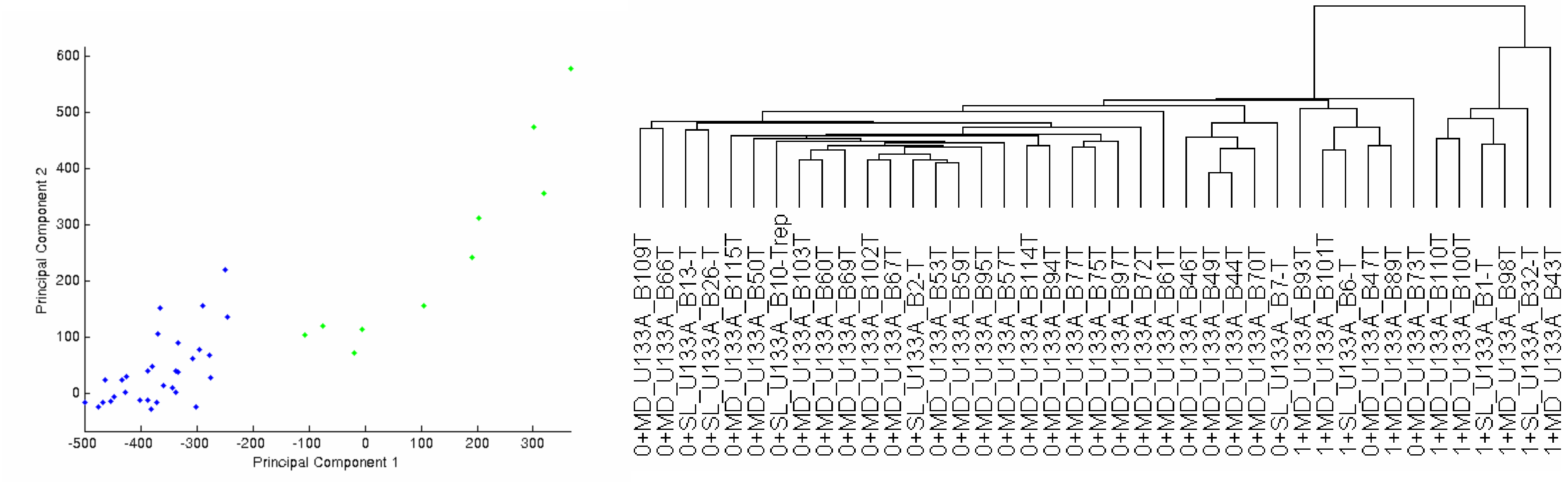

Figure S3: ***PCA and Hierarchical Clustering on Doane et al. Cohort filtered for 400-gene signature by Farmer et al.*** The 400-gene signature was identified by Farmer et al. to discriminate the molecular apocrine from the basal phenotype. The original data as normalized by Doane et al. was filtered for the 400-gene signature. **(LEFT)** PCA plot of the filtered data. The hypothesized "molecular apocrine" phenotype is represented by the color green and "non-molecular apocrine" phenotype by the color blue. **(RIGHT)** Dendrogram from Hierarchical Clustering of the filtered data. Phenotypes of "molecular apocrine" or "non-molecular apocrine" are indicated by 1 and 0, respectively, preceding the sample ID. Clustering was performed by Pairwise-Average Linkage using Euclidian Distance.

Figure S4

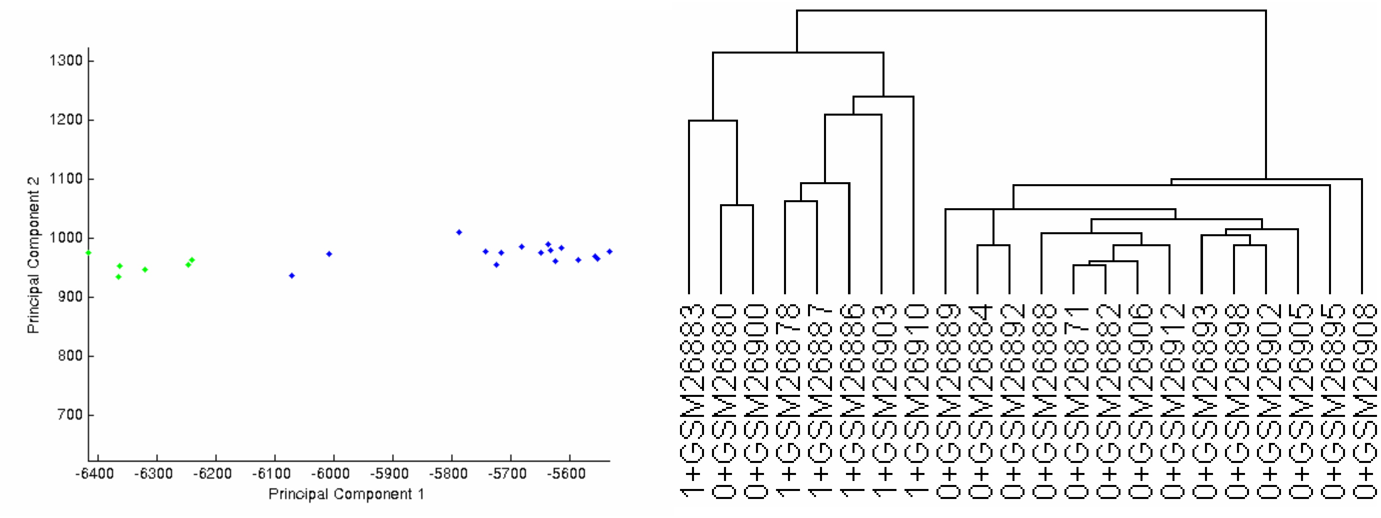

Figure S4: ***PCA and Hierarchical Clustering on Farmer et al. Cohort filtered for 138-gene signature by Doane et al.*** The 138-gene signature was identified by Doane et al. to discriminate the molecular apocrine other ER^- tumors. The original data normalized by Farmer et al. was filtered for this 138-gene signature. **(LEFT)** PCA plot of the filtered data. The hypothesized “molecular apocrine” phenotype is represented by the color green and “non-molecular apocrine” phenotype by the color blue. **(RIGHT)** Dendrogram from Hierarchical Clustering of the filtered data. Phenotypes of “molecular apocrine” or “non-molecular apocrine” are indicated by 1 and 0, respectively, preceding the sample ID. Clustering was performed by Pairwise-Average Linkage using Euclidian Distance.

Figure S5

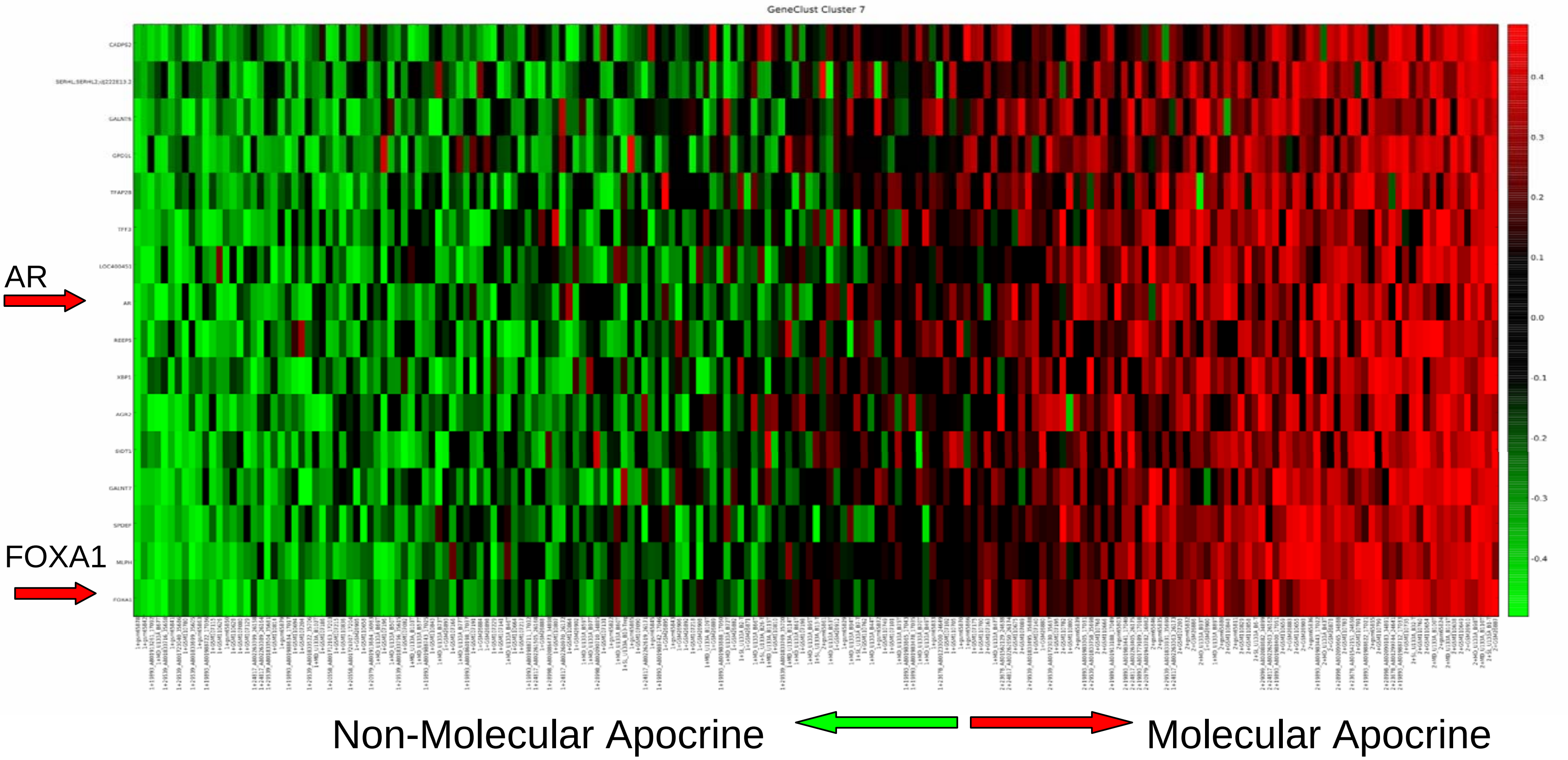

Figure S5: ***Heat-map of Gene Cluster 7 Identified by Unsupervised Gene Shaving.*** This gene cluster 7 includes both the AR and FOXA1 genes. Phenotypes of "molecular apocrine" or "non-molecular apocrine" are indicated by 2 and 1, respectively, preceding the sample ID. Molecular apocrine samples segregate with higher expression of the genes in this cluster.

Figure S6

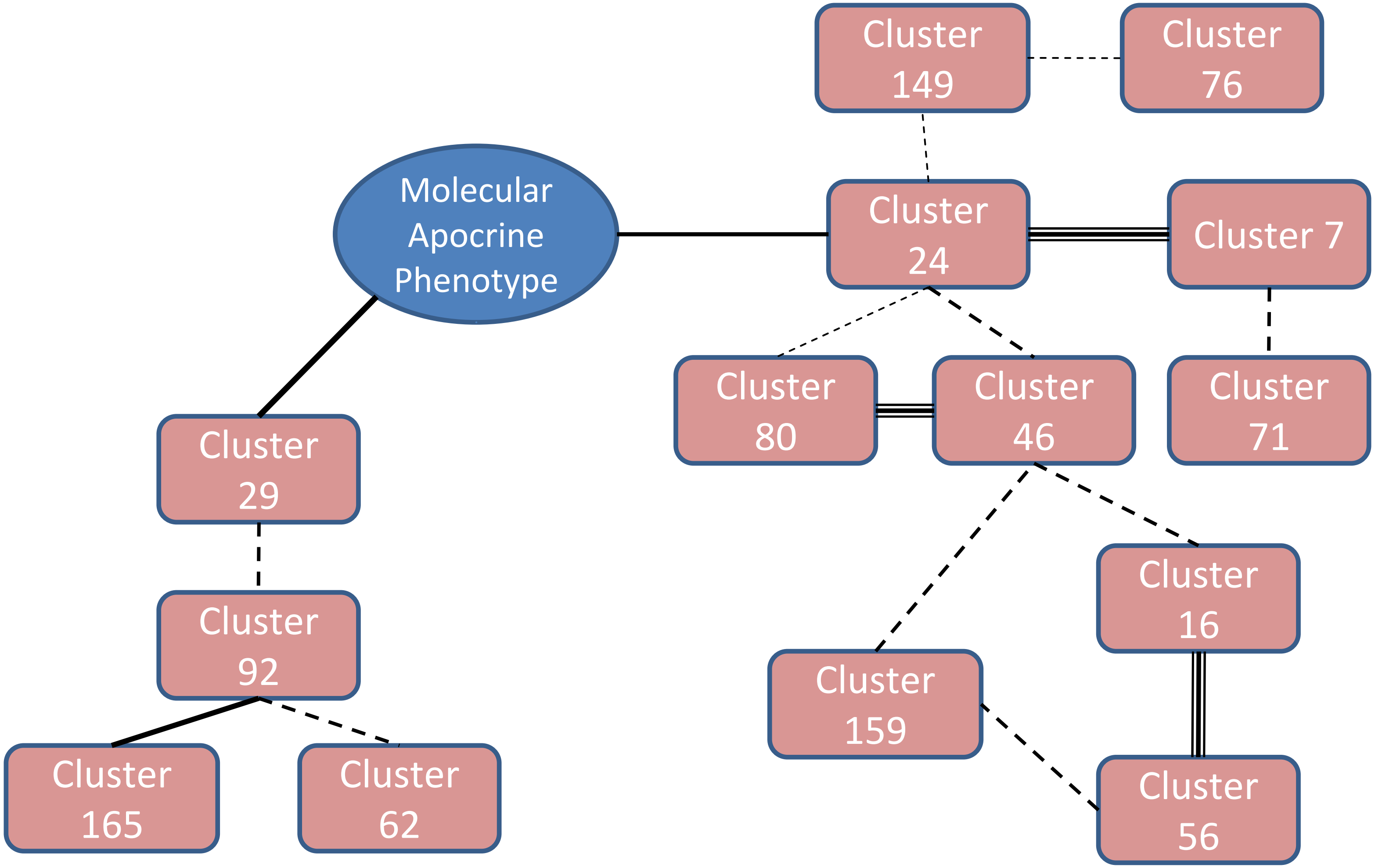

Figure S6: *Robust Bayesian Network Analysis of Top Molecular Apocrine-related Gene Clusters Identified Through Gene Shaving*. Robust Bayesian Network Analysis postulates network interactions between the top apocrine-related gene clusters, with the strength of relationships indicated by boldness of links. The 14 interacting clusters are depicted along with a node illustrating the "molecular apocrine phenotype." Links to the phenotype node indicate clusters with the strongest association with the molecular apocrine phenotype.

Figure S7

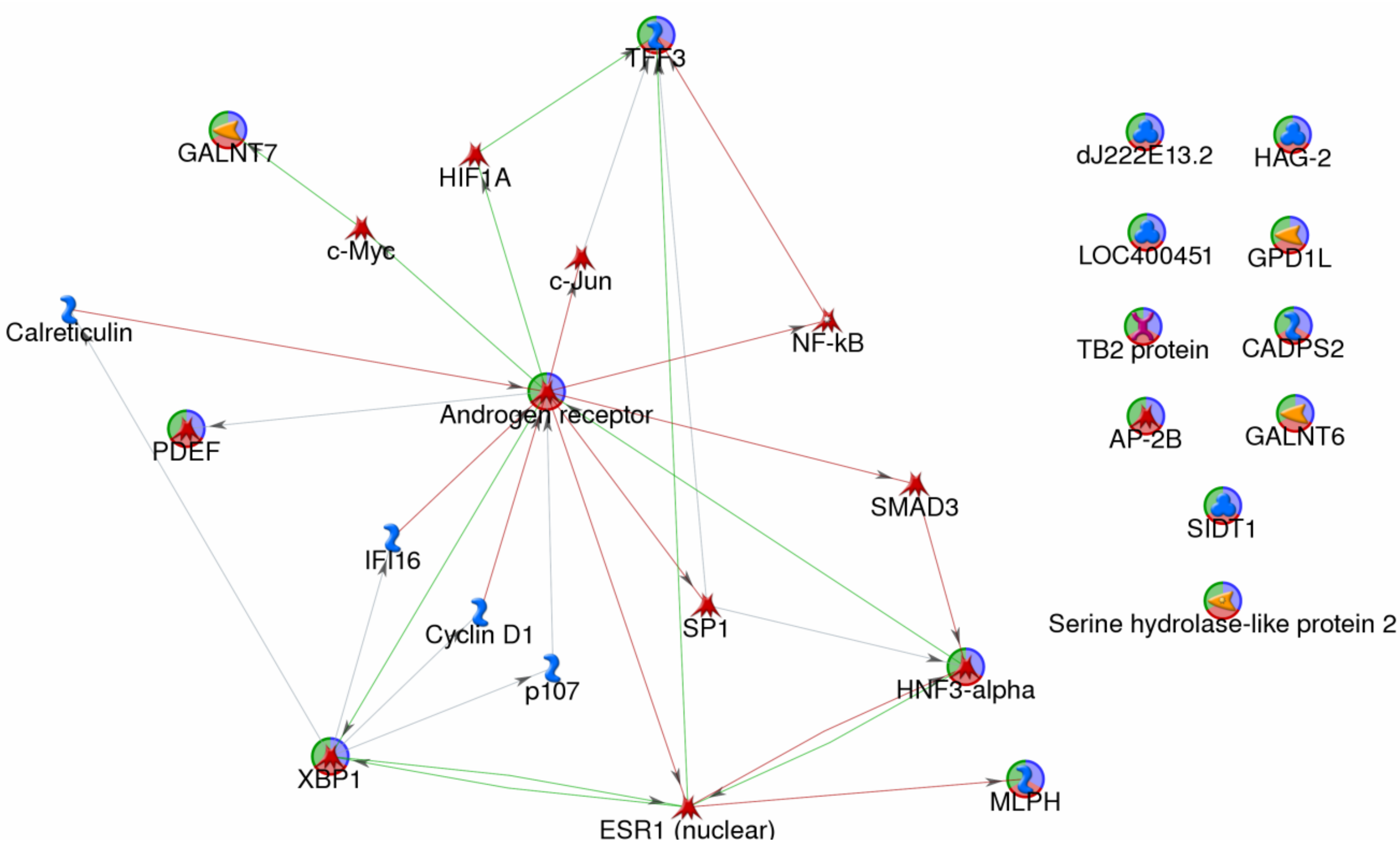

Figure S7: Connectivity within *Cluster 7 from Metacore.* Gene symbols with superimposed circles are members of the cluster. Graph is generated using Dykstra's algorithm and allowing up to three intervening nodes between members of the cluster.

Figure S8

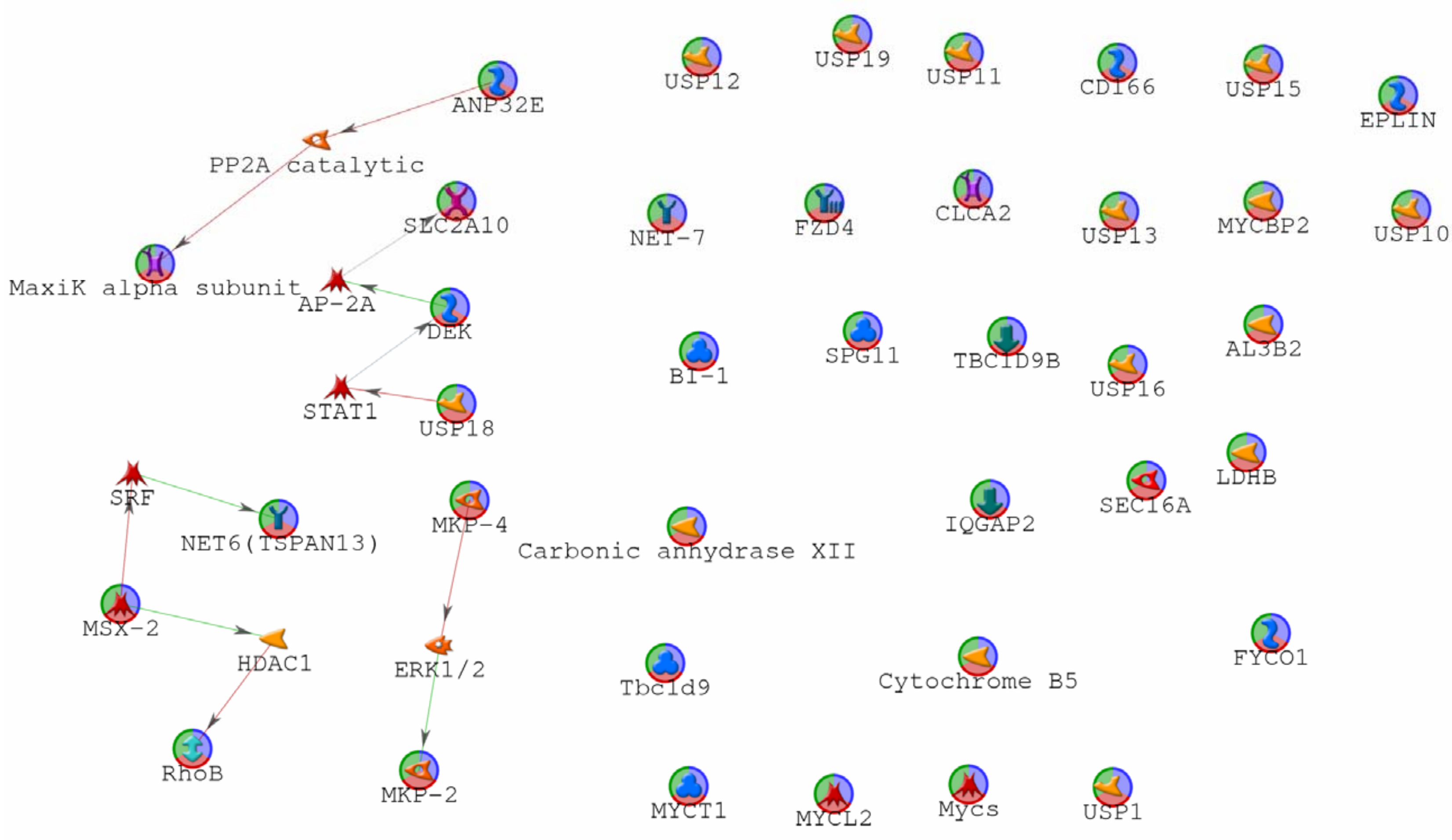

Figure S9: Connectivity within *Cluster 24 from Metacore.* Gene symbols with superimposed circles are members of the cluster. Graph is generated using Dykstra's algorithm and allowing up to three intervening nodes between members of the cluster.

Figure S9

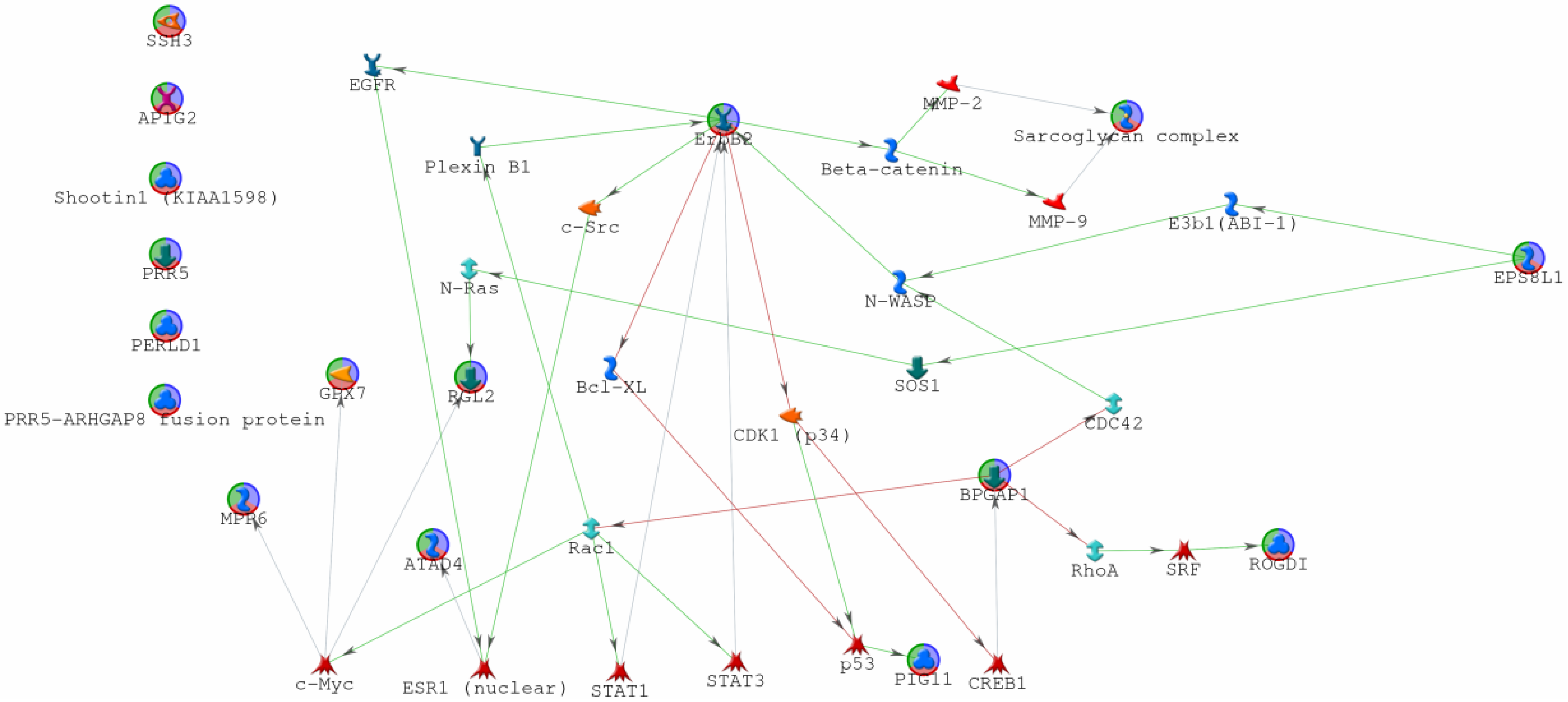

Figure S10: Connectivity within *Cluster 71 from Metacore.* Gene symbols with superimposed circles are members of the cluster. Graph is generated using Dykstra's algorithm and allowing up to three intervening nodes between members of the cluster.

Figure S10

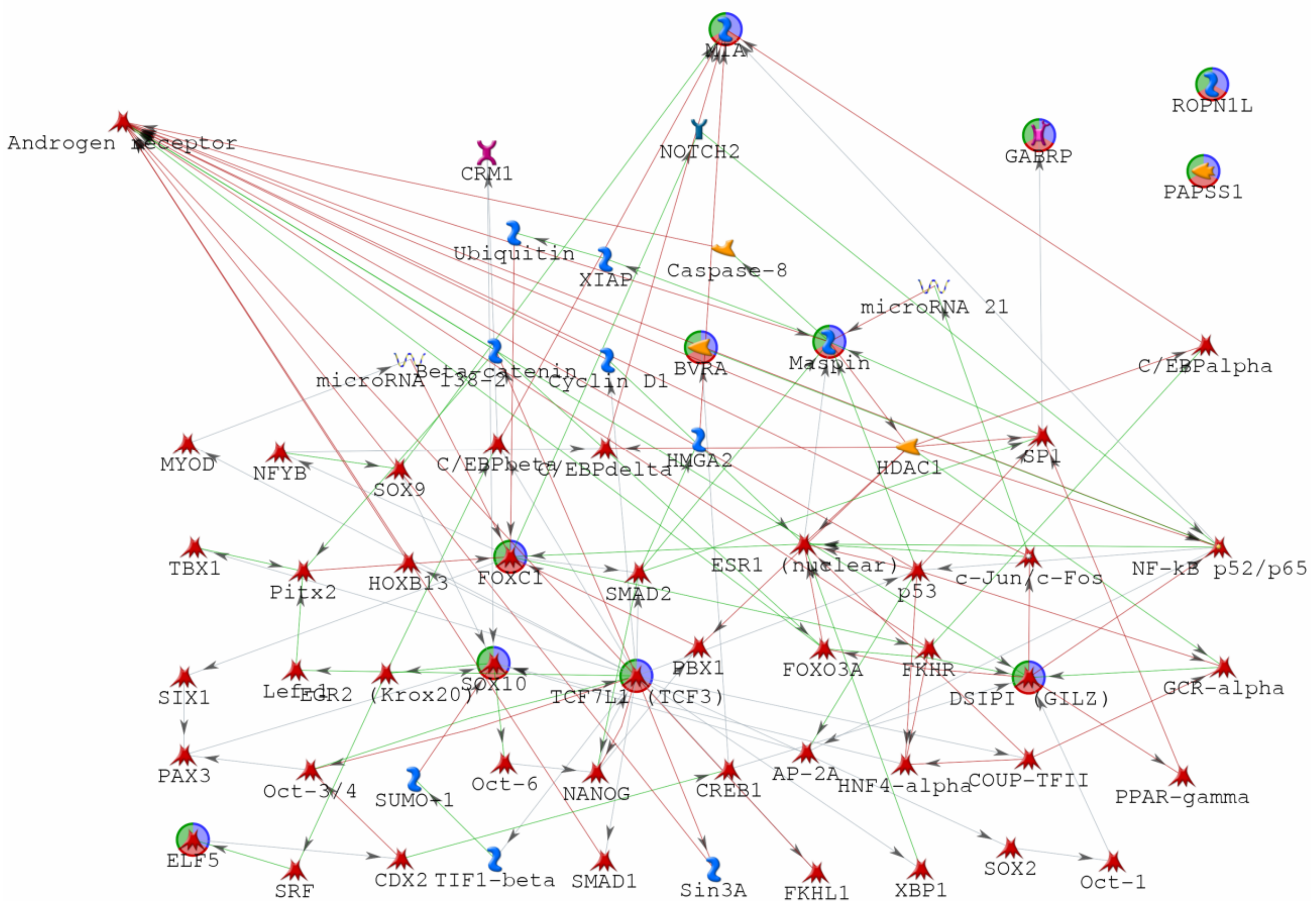

Figure S8: Connectivity within *Cluster 16 from Metacore.* Gene symbols with superimposed circles are members of the cluster. Graph is generated using Dykstra's algorithm and allowing up to three intervening nodes between members of the cluster.
